# Circulating intestine-derived exosomal miR-328 in plasma, a possible biomarker for estimating BCRP function in the human intestines

**DOI:** 10.1038/srep32299

**Published:** 2016-08-30

**Authors:** Keisuke Gotanda, Takeshi Hirota, Jumpei Saito, Masato Fukae, Yu Egashira, Noritomo Izumi, Mariko Deguchi, Miyuki Kimura, Shunji Matsuki, Shin Irie, Ichiro Ieiri

**Affiliations:** 1 Department of Clinical Pharmacokinetics, Graduate School of Pharmaceutical Sciences, Kyushu University, Fukuoka, Japan; 2 SOUSEIKAI Global Clinical Research Center, Fukuoka, Japan

## Abstract

A variant in the breast cancer resistance protein (BCRP) gene, 421C> A is a useful biomarker for describing large inter-individual differences in the pharmacokinetics of sulfasalazine (SASP), a BCRP substrate. However, large intra-genotypic variability still exists in spite of the incorporation of this variant into the pharmacokinetics of SASP. Since miR-328 negatively regulates BCRP expression in human tissues, we hypothesized that exosomal miR-328 in plasma, which leaks from the intestines, is a possible biomarker for estimating BCRP activity in the human intestines. We established an immunoprecipitation-based quantitative method for circulating intestine-derived miR-328 in plasma using an anti-glycoprotein A33 antibody. A clinical study was conducted with an open-label, non-randomized, and single-arm design involving 33 healthy participants. Intestine-derived exosomal miR-328 levels positively correlated (*P* < 0.05) with SASP AUC_0-48_, suggesting that subjects with high miR-328 levels have low intestinal BCRP activity, resulting in the high AUC of SASP. Circulating intestine-derived exosomal miR-328 in plasma has potential as a possible biomarker for estimating BCRP function in the human intestines.

The ATP-binding cassette transporter family member breast cancer resistance protein (BCRP, *ABCG2*) is one of the most important intestinal efflux transporters involved in the intestinal absorption or permeability of drugs. In the human duodenum, BCRP is expressed at the same high levels as P-glycoprotein (P-gp)^1^. BCRP and P-gp engage in the extraction of their substrates back into the intestinal lumen, which may lead to the poor absorption and low oral bioavailability of their substrates. Sulfasalazine (SASP) and statins have been identified as substrates of BCRP with low oral bioavailabilities^2^,^3^. Hence, BCRP in the intestines is considered to play a crucial role in the oral bioavailability, disposition, and tissue protection of BCRP substrates. Previous studies indicated that BCRP expression levels and the pharmacokinetics of BCRP substrates were affected by *ABCG2* polymorphisms^4^,^5^. The *ABCG2* non-synonymous genetic variant, 421C>A (rs2231142, 141Q>K) is a useful indicator for describing large inter-individual differences in the bioavailability of SASP, particularly in its role in intestinal absorption, principally because SASP is not metabolized in the human liver^5^,^6^. We previously reported significant differences (*P* < 0.05) in the area under the plasma concentration versus time curve (AUC) of SASP in subjects with different *ABCG2* genotypes^5^. The mean (±SD) AUC_0–48_ of SASP in wild-type homozygotes (421C/C), heterozygotes (421C/A), and mutant homozygotes (421A/A) were 171 ± 85, 330 ± 194, and 592 ± 275 μg·h/ml, respectively. However, large intra-genotype variability in AUC_0-48_ still exists in spite of the incorporation of 421C>A into the stratification^5^. Inter-individual differences in the pharmacokinetics of SASP are not fully explained by the 421C>A *ABCG2* variant.

MicroRNAs (miRNAs) also regulate the expression of BCRP in the human placenta^7^. MiR-328 expression level have been negatively correlated with BCRP mRNA and protein levels^7^. MiRNAs are small non-coding RNAs that control post-transcriptional gene expression and are involved in human physiological processes. Several recent studies showed that miRNAs are secreted into human body fluids such as plasma and urine from tissues and epithelial cells^8^,^9^,^10^,^11^. Circulating miRNAs in plasma are packed into exosomes, and exosomal miRNAs are resistant to biochemical degradation by ribonucleases^12^. Thus, circulating miRNAs have recently been recognized as biomarkers for the non-invasive diagnosis of various tumor entities^9^. Since exosomal miRNA contents largely reflect cellular miRNA contents^13^, exosomal miRNA levels in plasma may be used as a measure of the expression levels of original cellular miRNAs. Exosomes are nano-sized membrane vesicles (<100 nm in diameter) and are produced by a double inversion of the plasma membrane through the process of endocytosis^14^. The protein content and orientation of proteins in the exosomal membrane reflect those in the plasma cell membrane of the originating cell^14^. Therefore, the origin of exosomes may be identified through specific proteins in the membrane of the originating cell. Based on these findings, we established a method to isolate intestine-derived exosomal miRNAs from plasma using immunoprecipitation with a monoclonal antibody against human glycoprotein A33 (GPA33), which is specifically expressed in the normal human small intestine and colonic epithelium^15^,^16^.

In the present study, we measured the levels of intestine-derived exosomal miRNAs in plasma, and evaluated relationships between circulating miRNA levels and SASP AUCs (as an indicator of intestinal BCRP activity). We also determined whether intestine-derived exosomal miR-328 has potential as a candidate biomarker for predicting the functional activity of BCRP in the human intestines.

## Results

No clinically undesirable signs and symptoms possibly attributed to the administration of Sulfasalazine were recognized throughout the study. All subjects completed the study successfully according to the protocol.

### SASP pharmacokinetics

The mean (±SD) AUC_0–48_ and mean peak plasma concentration (C_max_) of SASP was 299 ± 186 μg·h/ml and 23.5 ± 12.7 μg/ml. The mean apparent clearance (CL_total_/F) was 9.1 ± 5.3 L/h. These results are consistent with our previous findings^5^.

### Extraction of intestine-derived exosomes from plasma by immunoprecipitation using an anti-GPA33 antibody

**Figure 1:**
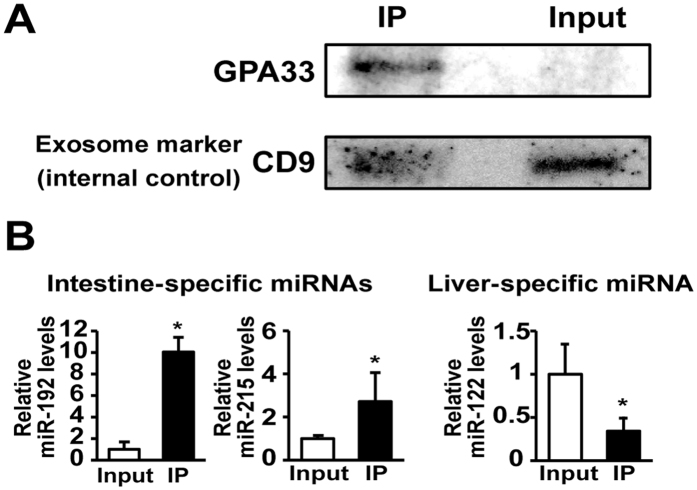
Immunoprecipitation experiments confirming the identification of intestine-derived exosomes. (**A**) GPA33 proteins in total exosomes and intestine-derived exosomes were detected by Western blotting. CD9 is a marker of exosomes. (**B**) Tissue-specific miRNA levels in intestine-derived exosomes. MiR-192 and miR-215 are intestine-specific miRNAs and miR-122 is a liver-specific miRNA. Relative miRNA levels were determined by qRT-PCR. Each column represents the mean ± S.D. (n = 3). *P < 0.05, significantly different from the input. Input: total exosome samples, IP: Immunoprecipitated samples.

GPA33 is specifically expressed in the normal human small intestine and colonic epithelium^16^. In order to confirm that the origin of immunoprecipitated exosomes using an anti-GPA33 antibody is the intestines, we measured GPA33 and intestine-specific miRNA (miR-192 and miR-215) levels in exosomes^17^,^18^. GPA33, which was normalized with CD9 (an exosomal marker)^19^, was concentrated via immunoprecipitation (Fig. 1A). CD9 was highly enriched (>10 fold) in total exosomes compared with total cells^19^,^20^. In additionally, Mallegol *et al*. reported that intestine-derived exosomes showed expression of CD9 at apical and basolateral membranes as well as GPA33^21^. These results suggest that CD9 is reasonable exosomal marker in intestine-derived exosomes. Intestine-specific miR-192 and miR-215 levels in immunoprecipitated exosomes were significantly higher (approximately 10- and 3-fold, respectively) than those in total plasma exosomes (*P* < 0.05, Fig. 1B). In contrast, liver-specific miR-122 levels in immunoprecipitated exosomes were significantly lower than those in total plasma exosomes (*P* < 0.05, Fig. 1B). These results indicate that the origin of immunoprecipitated exosomes by the anti-GPA33 antibody is the intestines.

### MiR-328 in intestine-derived exosomes

**Figure 2:**
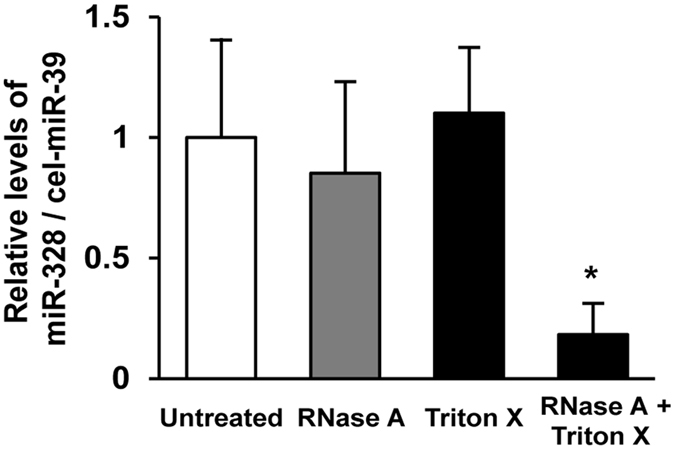
MiR-328 in intestine-derived exosomes. Comparisons of the means for multiple groups against the non-treatment control were analyzed with Tukey–Kramer’s multiple comparison tests (*P < 0.01). Each column represents the mean ± S.D. (n = 3).

In order to confirm whether miR-328 is packed into GPA33-immunoprecipitated exosomes, we examined exosomal miR-328 levels in GPA33-immunoprecipitated samples in the presence of a detergent (1% Triton X-100), RNase A, or both using qRT-PCR. MiR-328 levels in the samples treated with the detergent or RNase A were similar to those in untreated immunoprecipitated samples (Fig. 2). In contrast, miR-328 levels were significantly decreased (*P* < 0.01) after the treatments with 1% Triton X-100 and RNase A. Transmission electron microscopy images showed that <100 d.nm particles existed in GPA33-immunoprecipitated samples, but not in that after the treatment with 1% Triton X-100 (Supplementary Information 1). These results indicate that miR-328 is packaged in intestine-derived exosomes.

### Determination of the most stable reference miRNAs for the normalization of miR-328 measurements

**Figure 3:**
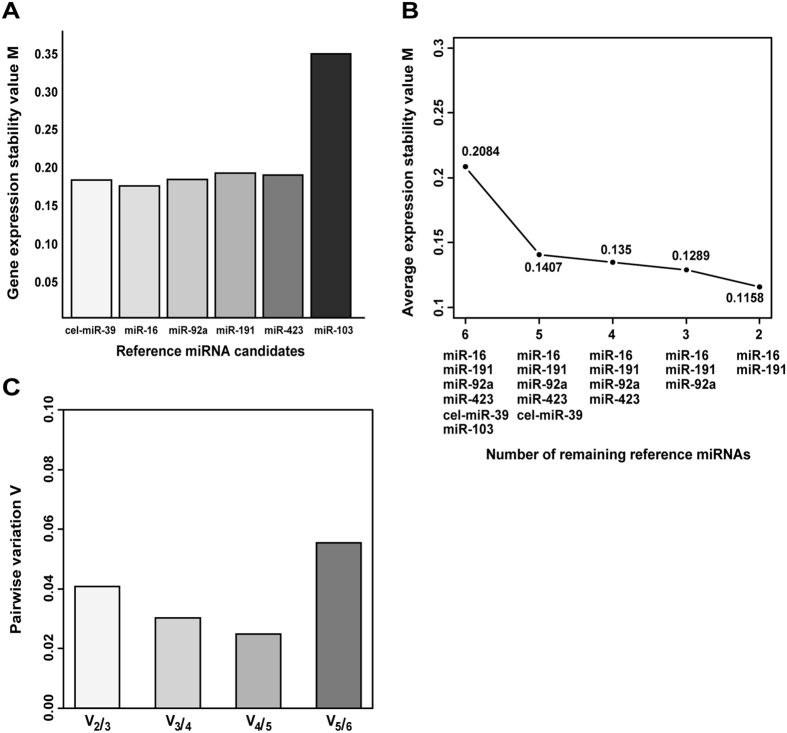
Gene expression stability and pairwise variations in candidate reference miRNAs. (**A**) In order to determine the optimum number of reference miRNAs, miRNA level stability measures (M) were calculated for all candidate miRNAs. (**B**) The average of stability M values in a stepwise exclusion of the least stable reference miRNA. (**C**) Determination of the optimal number of miRNAs for normalization. Average pairwise miRNA level variations in each miRNAs compared to all other evaluated miRNA pairwise variation (V_n/n+1_) were calculated using the geNorm algorithm provided by R and Bioconductor. A pairwise variation of 0.15 was accepted as the cut-off value, less than which the inclusion of additional reference miRNA was not necessary for reliable normalization.

The expression of candidate references was evaluated using the geNorm analysis in order to identify a set of reliable reference miRNAs for a miR-328 relative expression analysis. The six candidate reference miRNAs (miR-16, miR-92, miR-103, miR-191, miR-423-5p, and cel-miR-39) were selected according to previous studies^18^,^22^,^23^,^24^. Threshold cycle values of endogenous candidate miRNAs and miR-328 were shown in Supplementary Information 2. GeNorm provides a ranking of 6 reference miRNAs based on the reference miRNA stability measure M. The M value for each miRNA was similar, indicating that the expression of the 5 miRNAs had similar variations (Fig. 3A,B). Pairwise variations (V_n/n+1_) allow for the identification of the optimal number of reference miRNAs. Since pairwise variations in all combination patterns were less than 0.15 (the cut-off value^25^), we selected the recommended minimal number of references, 3 miRNAs (miR-16, miR-92a, and miR-191) as a set of stable reference miRNAs (Fig. 3C).

### Relationship between miR-328 levels in intestine-derived exosomes in plasma and SASP AUC_0-48_

**Figure 4:**
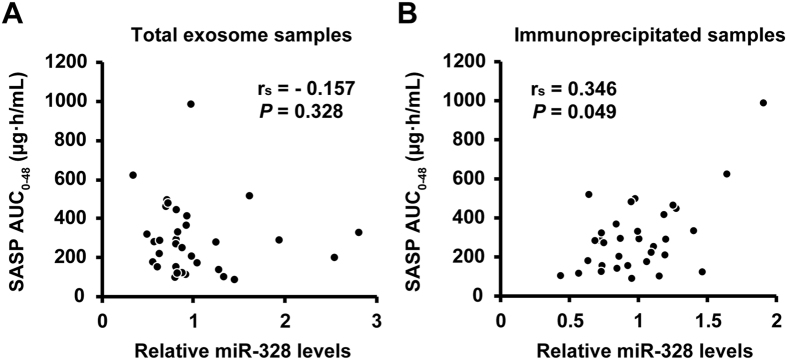
Relationship between miR-328 levels in total exosomes or intestine-derived exosomes in plasma and SASP AUC_0-48_. MiR-328 levels were normalized with the most stable reference genes selected by geNorm for all samples. Significance was determined by Spearman’s correlation test.

In order to investigate whether total plasma exosomal miR-328 and intestine-derived exosomal miR-328 are applicable as candidate biomarkers for predicting the functional activity of BCRP in the intestines, the relationships between miR-328 levels and SASP AUC_0-48_ were analyzed. Total plasma exosomal miR-328 levels did not correlate with SASP AUC_0-48_ (r_s_ = −0.157, *P* = 0.382, Fig. 4A), whereas the intestine-derived exosomal miR-328 levels positively correlated with SASP AUC_0-48_ (r_s_ = 0.346, *P* < 0.05, Fig. 4B). In addition, the intestine-derived exosomal miR-328 levels which were normalized to cel-miR-39 spike-in correlated with SASP AUC_0-48_ (r_s_ = 0.358, *P* < 0.05, Supplementary Information 3). MiR-328 levels in intestine-derived exosomes did not correlate with miR-328 levels in total exosomes (*P* = 0.572, Supplementary Information 4).

## Discussion

The aim of the present study was to determine whether total plasma and intestine-derived exosomal miR-328 levels are candidate biomarkers for predicting the functional activity of BCRP in human the intestines. We attempted investigate the relationship between exosomal miR-328 levels and SASP AUC_0-48_ as a phenotype of intestinal BCRP activity. The results obtained indicated that no correlation existed between SASP AUC_0-48_ and miR-328 levels in total plasma exosomes (Fig. 4A). Circulating miRNAs isolated from total plasma exosomes are considered to be derived from various human tissues, which, in turn, results in total plasma exosomal miR-328 consisting of non-specific tissue-derived miR-328. Since intestinal BCRP plays an important role in the pharmacokinetics of SASP, intestine-derived miR-328 is expected to be a more accurate biomarker. It is difficult to obtain the blood and the intestinal cells form the same individual. Instead of evaluating the correlation between the expression of miR-328 in the intestinal cells and circulating miR-328 levels, we showed secreted exosomal miR-328 levels reflect endogenous miR-328 levels in Caco-2 cells. These results suggest that expression of miR-328 in the intestinal cells is correlated with circulating miR-328 levels (Supplementary Information 5). Although there is no currently well-established method for isolating specific tissue-derived exosomes, immunoprecipitation with an antibody to the GPA33 protein, which is specifically expressed in intestinal epithelial cells, was developed in order to isolate intestine-derived exosomes in plasma. This is the first attempt to isolate intestine-derived exosomes.

We measured tissue-specific miRNAs; miR-192 and miR-215 for the intestine, and miR-122 for the liver^17^,^18^, to confirm that immunoprecipitated samples are derived from the intestines. The expression of the GPA33 protein was also measured in these samples. As shown in Fig. 1B, intestine-specific miR-192 and miR-215 levels and hepatic miR-122 levels in the immunoprecipitated samples were approximately 10-fold and 3-fold higher, respectively, and also significantly lower than those in total plasma exosomes. The GPA33 protein was also detected only in immunoprecipitated samples (Fig. 1A). These results support current immunoprecipitation processes using a GPA33 antibody successfully extracting intestine-derived exosomes in plasma.

The next important issue in this study is that miR-328 was actually packed into intestine-derived exosomes. Therefore, immunoprecipitated samples were treated with a detergent, RNase A, or both. MiR-328 levels in the samples treated with the detergent or RNase A were similar to those in untreated immunoprecipitated samples, while miR-328 levels in the immunoprecipitated samples treated with the detergent and RNase A were significantly decreased (Fig. 2), suggesting that miR-328 is packaged in intestine-derived exosomes.

It is important to note the analytical issue of appropriate normalization strategies for the quantification of exosomal miRNAs. MiRNA expression profiles may differ in various tissues. Although small nuclear RNAs (snRNAs), such as RNU6B, RNU44, and RNU48, are used as reference genes to normalize the expression levels of cellular miRNAs, exosomal miRNAs are circulating cell-free miRNAs; therefore, snRNAs are not considered suitable as internal references^26^,^27^. Stably expressed miR-16 is currently used as a normalization control in quantitative miRNA expression analyses^23^. Other researchers have adapted normalization strategies based on the quantification of spike-in non-human synthetic miRNAs^24^. Several recent studies used the geNorm algorithm for the accurate normalization of miRNA expression^20^. This algorithm evaluates the relative expression stability (M value) of a number of candidate internal reference genes and measures normalization factors (NF) from selected candidate genes^25^. Appropriate reference genes must not only correct technical differences in quantification, but also reduce the influence of pre-analytical variations as much as possible. Hence, we used the geNorm algorithm to quantify exosomal miR-328 in the present study. Our candidate internal reference miRNAs (miR-16, miR-92a, miR-103, miR-191, and miR-423-5p) have often been used in previous studies^18^,^22^,^23^,^24^. Three miRNAs (miR-16, miR-92a, and miR-191) were selected by the geNorm algorithm because their expression levels in all of our intestine-derived exosomes samples were stable (Fig. 3).

Our study focused on the relationship between intestine-derived exosomal miR-328 levels and intestinal BCRP activity. Some *in vitro* studies have shown that miR-328 inhibits the expression of BCRP mRNA and protein^28^,^29^. We previously demonstrated that miR-328 levels negatively correlated with BCRP mRNA and protein expression levels in the human placenta^7^. In addition, we reported that the expression levels of miR-328 were regulated by an epigenetic mechanism^7^. The methylation patterns of several CpG dinucleotides proximal to two C/EBPα-binding sites in the miR-328 5′-flanking region negatively correlated with miR-328 levels, and positively correlated with BCRP protein levels in human placental samples^7^. Other miRNAs such as miR-519c, miR-520h, and miR-212 also regulate the expression of BCRP^28^,^29^. Li *et al*. reported that miR-519c and miR-328 exerted stronger effects on the regulation of BCRP expression in human breast cancer cells than miR-520h^28^. Furthermore, epigenetic regulation may be involved in the expression of miR-519c and miR-520h. Suzuki *et al*. re-expressed these miRNAs in gastric cancer cell lines after a treatment with 5-aza-2-deoxycytidine as a DNA hypomethylating agent and 4-phenylburtyrate as a histone deacetylase inhibitor^30^. The expression of these miRNAs was increased by both treatments. However, according to Liang *et al*., the expression of these miRNAs is absent or very low in the normal colorectal mucosa or small intestine^18^. Therefore, further studies are needed in order to determine whether these miRNAs and as yet undiscovered miRNAs regulate the expression of BCRP in human tissues.

The results of the present study suggest that intestine-derived exosomal miR-328 from plasma is potential biomarker for predicting the functional activity of BCRP in the intestines. In our previous study, significant differences in the AUC of SASP were observed among subjects with different *ABCG2* genotypes. As we described above, large intra-genotype variability in AUC_0-48_ still exists in spite of the incorporation of 421C>A into the stratification^6^. Specific tissue-derived miR-328 in plasma is an individual continuous value, and, thus, is expected to overcome this disadvantage of SNP stratification. However, since no concrete conclusions were reached regarding the relationship between miR-328 levels and AUC of SASP, further studies with a larger number of participants are needed in order to improve the precision of BCRP *in vivo* functions.

## Methods

### Subjects

A clinical study was conducted with an open-label, non-randomized, and single-arm design involving 33 healthy participants (age: 20–24 years; weight: 43.3–68.6 kg; BMI: 17.6–24.3 kg/m^2^). The *NAT2* genotype was prescreened, and slow acetylators (*5/*5, *6/*6, *7/*7) were excluded in order to avoid the adverse effects of SASP. Each subject was physically normal and had no history of significant medical illness or hypersensitivity to any drugs. None had taken any drugs for at least 1 week before the study. This clinical study was approved by the Ethics Review Boards of Kyushu University (523-00) and Kyushu Clinical Pharmacology Research Clinic (1248CP), and written informed consent was obtained from all participants before the study. This study was registered in the UMIN Clinical Trials Registry (JPRN-UMIN000009976, February 22, 2013). The methods in this study were carried out in accordance with the approved guidelines.

### Clinical study design

A single oral dose of sulfasalazine (2,000 mg, 4 conventional tablets of salazopyrin, Pfizer, Tokyo, Japan) was administered after a 12-h overnight fast. Blood samples were drawn before dosing and 0.5, 1, 2, 3, 4, 6, 9, 12, 24, 36, and 48 h after dosing. Blood samples were centrifuged to produce plasma, and stored at −20 °C until quantified.

### Genotyping

Genomic DNA was extracted from EDTA-treated whole blood samples using the Toyobo blood kit on a Toyobo HMX-2000 robot (Toyobo, Osaka, Japan). Extracted DNA was genotyped for known SNPs in *NAT2* using TaqMan SNP Genotyping Assays (Applied Biosystems, Foster City, CA, USA), as described previously^4^. The following SNPs were genotyped: *NAT2* 341T>C (rs1801280), 590G>A (rs1799930), and 857 G>A (rs1799931).

### Quantification of sulfasalazine

Sulfasalazine concentrations in plasma were measured using high-performance liquid chromatography (HPLC) as previously described with minor modifications^4^. Briefly, a 100-μl plasma sample containing the internal standard (0.125 μg/ml of p-dimethylaminobenzaldehyde) was added to 300 μl of methanol. The mixture was stored on ice for 10 min, centrifuged at 10,000 × *g* for 2 min, and the supernatant was then passed through a microporous membrane filter (Millex-GV 0.22-μm filters, Millipore Corp., Bedford, MA, USA). Each 20-μl sample was subjected to a HPLC analysis. A variable wavelength ultraviolet detector was adjusted to 365 nm for sulfasalazine. The assay was carried out based on the peak area ratios of sulfasalazine to the internal standard. The calibration curves were linear over a concentration range of 0.1 to 80 μg/ml (r^2^ > 0.999).

### Isolation of exosomes

Exosomes were extracted from 200 μl plasma before the administration of SASP using ExoQuick (System Biosciences, San Francisco, CA, USA). In order to obtain total plasma exosomes, plasma was centrifuged again at 15,000 × *g* for 15 minutes in order to remove cells and cellular fragments, and the subsequent filtration of plasma was accomplished through a microporous membrane filter (Millex-LG 0.20 μm, Millipore, Bedford, MA, USA). ExoQuick was added to the filtrate, and the sample was stored at 4 °C for 30 min. Exosome pellets collected by centrifugation at 1,500 × *g* for 30 minutes were suspended in 200 μL phosphate-buffered saline (PBS). Qinyu *et al*. characterized the isolated vesicles by ExoQuick^TM^^31^. The result of analysis by using Grainsize Analyzer showed that two peaks were observed in the vesicles, and the size-average was 48.42 d.nm; the sizes of the two peaks were 42.40 ± 25.98 and 126.9 ± 58.3 nm with weighted intensity of 68.2% and 31.8%, respectively. TEM examination revealed that the size of the vesicles was <200 nm, suggesting that the isolated vesicles by ExoQuick^TM^ are exosomes.

### Immunoprecipitation with magnetic beads

An anti-GPA33 antibody [EPR4240] (ab108938, Abcam, Cambridge, MA, USA) was conjugated to magnetic Dynabeads M-270 Epoxy (Invitrogen Life Technologies, Carlsbad, CA,USA). Dynabeads (1 mg) were incubated at 4 °C for 16 h on a rotor in the anti-GPA33 antibody (5 μL) and C1 (45 μL) and C2 (50 μL) mix buffers, which are components of the Dynabeads Antibody Coupling Kit (Invitrogen Life Technologies). After the beads had been placed on a magnet for 1 min, the supernatant was discarded and the beads were washed three times with PBS. Antibody-coupled magnetic beads were added to the total plasma exosome suspension, and the mixture was incubated at 4 °C for 2 h on a rotor. The mixture was placed on a magnet again for 1 min, the supernatant was discarded, and the beads were washed four times with PBS. GPA33 antibody-coupled beads were incubated with 100 μl of PBS for miRNA extraction.

### Western blotting

Total plasma exosomes and GPA33-immunoprecipitated exosomes were diluted with RIPA buffer (50 mM Tris-HCl (pH 8.0), 150 mM NaCl, 0.5 w/v% sodium deoxycholate, 0.1 w/v% SDS, 1.0 w/v% NP-40, 1 mM EDTA) and Protease Inhibitor Cocktail (Sigma-Aldrich, St. Louis, MO, USA). In the glycosidase treatment, lysate samples were treated with 1 unit of N-glycosidase F (PNGase F, New England Biolabs, Ipswich, MA, USA) at 37 °C for 1 h. The treated lysate samples were separated on 11% SDS-polyacrylamide gels and transferred to polyvinylidene membranes. The membranes were hybridized with a rabbit monoclonal antibody against GPA33 (ab108938, Abcam) or with a mouse monoclonal antibody against CD9, which are the assumed exosome markers (CD9 antibody [MEM-61], GeneTex Inc., Irvine, CA, USA). Immunocomplexes were hybridized with an anti-rabbit or anti-mouse IgG horseradish peroxidase-linked whole antibody (GE Healthcare, Little Chalfont, Buckinghamshire, UK). Membranes were washed three times in 0.5% Tween-PBS, and specific bands were visualized using the Amersham ECL Select Western Blotting Detection Reagent (GE Healthcare) according to the manufacturer’s instructions.

### Detergent and RNase treatments

In order to confirm that miR-328 is packed into GPA33-immunoprecipitated exosomes, immunoprecipitated samples were treated with RNase A (20 ng/ml, Roche Diagnostics, Indianapolis, IN, USA), 1% Triton X-100 (Promega, Madison, WI, USA), or both at 37 °C for 10 min before miRNA extraction.

### miRNA extraction and quantification

Total RNAs (*i.e.*, mRNAs and miRNAs) were extracted from total plasma exosomes and GPA33-immunoprecipitated exosomes using the miRVana PARIS kit (Ambion Life Technologies, Carlsbad, CA, USA) according to the manufacturer’s instructions. Prior to phenol chloroform extraction, 25 fmol of exogenous synthetic cel-miR-39 as an external control to normalize miRNA detection was spiked into each sample. The amounts of miRNAs (cel-miR-39, miR-16, miR-92a, miR-103, miR-122, miR-191, miR-192, miR-215, miR-328, and miR-423-5p) were quantified in triplicate by qRT-PCR using the Universal cDNA Synthesis Kit and ExiLENT SYBR Green master mix (EXIQON, Woburn, MA, USA) or TaqMan MicroRNA Assay Kits according to the manufacturers’ protocols (Applied Biosystems). The relative quantity for miR-328 was determined by the comparative Ct method (2^–ΔΔCt^) or geNorm analysis.

### GeNorm analysis

The GeNorm analysis was performed with the statistical software program R (version 3.0.2, R Development Core Team, 2011) with Bioconductor packages (NormqPCR and SLqPCR, http://www.bioconductor.org/packages/release/bioc/html/(NormqPCR.html and SLqPCR.html)). Regarding each reference miRNA, the miRNA level stability measure (M) was defined as the average standard deviation of the log-transformed miRNA expression ratio for all other miRNA combinations. The step-wise elimination of a miRNA exhibiting the highest M value and the recalculation of a new M value for the remaining miRNAs was repeated until two miRNAs remained. Pairwise variations (V_n/n+1_) between two normalization factors (NF_n_ and NF_n+1_) using the geometric mean of the expression levels of N and N + 1 number of reference miRNAs were subsequently calculated, reflecting the effects of adding an (N + 1)th miRNA.

### Non-compartmental pharmacokinetic analysis

The pharmacokinetic parameters of SASP were calculated based on a non-compartmental analysis. C_max_ was obtained directly from the data. The AUC_0–48_ was calculated according to the linear trapezoidal rule. We calculated apparent oral clearance (CL_total_/F = Dose/AUC_0–48_).

### Statistical analyses

Statistical analyses were performed with the statistical software program R (R Development Core Team, 2011). The means for two groups were compared with an unpaired Student’s *t*-test (two-tailed). Comparisons of means for multiple groups against controls (untreated exosomes or pharmacokinetic parameters of wild-type homozygotes) were analyzed with Tukey–Kramer’s multiple comparison test. A 5% level of probability was considered to be significant. Relationships Correlation between variables were assessed using Spearman’s rank method.

## Additional Information

**How to cite this article**: Gotanda, K. *et al*. Circulating intestine-derived exosomal miR-328 in plasma, a possible biomarker for estimating BCRP function in the human intestines. *Sci. Rep.* **6**, 32299; doi: 10.1038/srep32299 (2016).

## Supplementary Material

Supplementary Information
