# Supplementary material for: Circulating intestine-derived exosomal miR-328 in plasma, a possible biomarker for estimating BCRP function in the human intestines
Source: Sci Rep. 2016 Aug 30;6:32299. doi: 10.1038/srep32299 (PMC5004159; doi:10.1038/srep32299)
Supplement: Supplementary Information [file srep32299-s1.doc]

**Supplementary materials:**

**Circulating intestine-derived exosomal miR-328 in plasma, a possible biomarker for estimating BCRP function in the human intestines.**

Keisuke Gotanda^1^, Takeshi Hirota^1^, Jumpei Saito^1^, Masato Fukae^1^, Yu Egashira^1^, Noritomo Izumi^2^, Mariko Deguchi^2^, Miyuki Kimura^2^, Shunji Matsuki^2^, Shin Irie^2^, Ichiro Ieiri^1*^

^1^Department of Clinical Pharmacokinetics, Graduate School of Pharmaceutical Sciences, Kyushu University, Fukuoka, Japan

^2^Kyushu Clinical Pharmacology Research Clinic (current name is SOUSEIKAI Sugioka Memorial Hospital Clinical Research Center), Fukuoka, Japan

**Supplementary Figure S1 Representative transmission electron microscopy images in GPA33-immunoprecipitated samples (A, C) and that after the treatment with 1% Triton X-100 (B, D).** TEM was performed to observe morphology of exosomes. (A, B: Magnification: ×200,000. Scale bars: 100 nm, C, D: Magnification: ×20,000. Scale bars: 1 μm). Samples (5 μL) were placed on a sheet of parafilm. A carbon-coated 400 mesh copper grid was floated on the drop for 1 minute and washed by a droplet of distilled water. The samples were negatively stained with 2% aqueous uranyl acetate and observed with a Hitachi H7600 TEM (Hitachi High-Technologies Corp., Tokyo, Japan) operated at 100 kV.


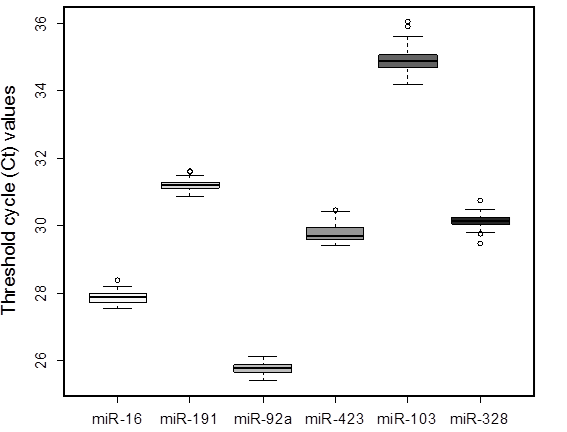


**Supplementary Figure S2 Ct values of intestine-derived exosomal miR-328 and candidate reference miRNAs.** Box plot of the distribution of CT values for miR-328 and candidate reference miRNAs shows the median values as lines across the box. Lower and upper boxes indicating the 25th percentile to the 75th percentile.


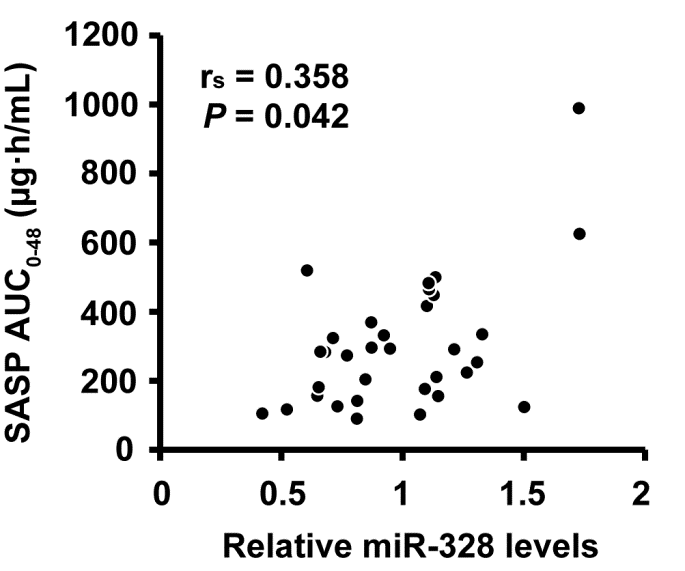


**Supplementary Figure S3 Relationship between SASP AUC_0-48_ and intestine-derived exosomal miR-328 levels normalized to cel-miR-39 spike-in.** Significance was determined by Spearman's correlation test. MiR-328 levels normalized to cel-miR-39 spike-in positively were correlated with SASP AUC_0-48_ (r_s_ = 0.358, *P* = 0.042, Figure S3).


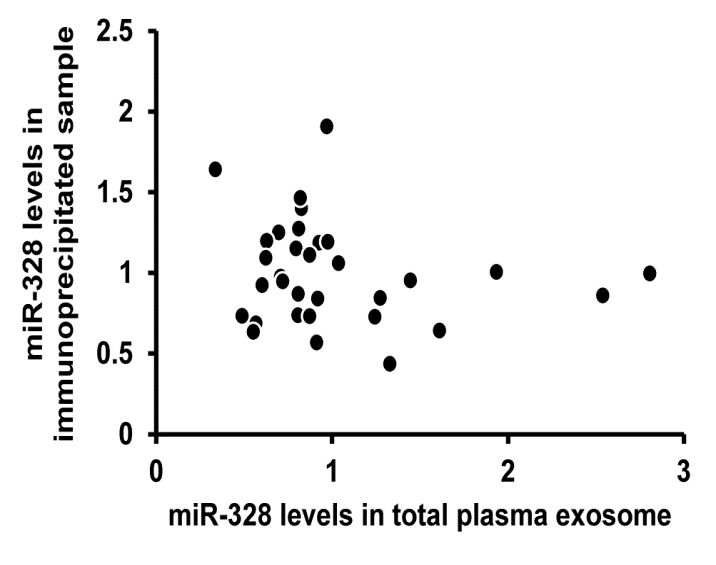


**Supplementary Figure S4 Relationship between intestine-derived exosomal miR-328 and total plasma exosomal miR-328 levels.** Significance was determined by Spearman's correlation test. MiR-328 levels in intestine-derived exosomes did not correlate with miR-328 levels in total plasma exosomes. (*P* = 0.572, Figure S4)


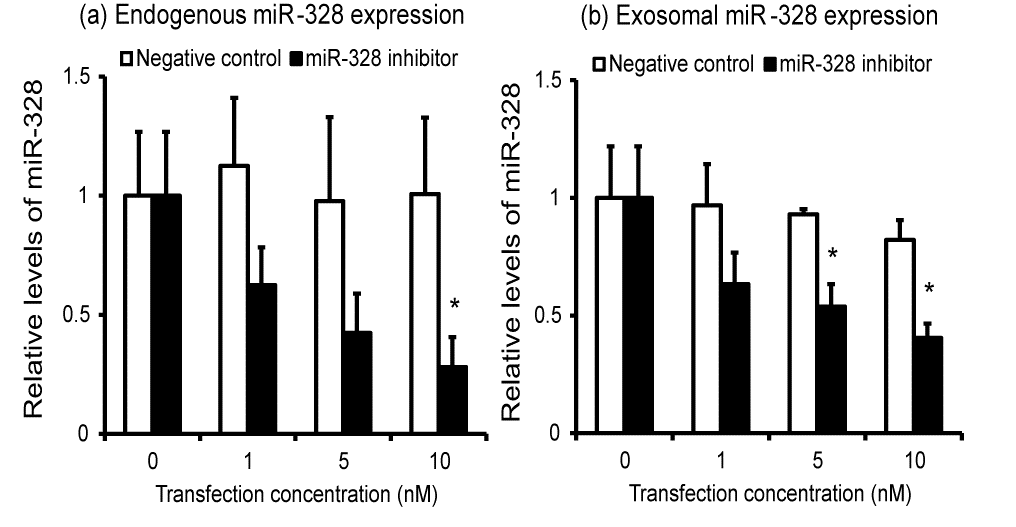


**Supplementary Figure S5 Quantification of total cellular miR-328 (A) and Caco-2 cell-derived exosomal miR-328 (B) levels in miR-328 inhibitor transfected Caco-2 cells.** Caco-2 cells were transfected with negative miRNA inhibitor and miR-328 inhibitor at three concentrations (1, 5, 10 nM). Relative miR-328 levels were determined by qRT-PCR and normalized to RNU6B (total cellular miR-328) and miR-16 (secreted Caco-2 cell-derived exosomal miR-328). Each column represents the mean ± S.D. (n=3). *, *P* < 0.05, significantly different from for the negative control (Tukey - Kramer's multiple comparison tests).
